# Comparative Validation of Five Quantitative Rapid Test Kits for the Analysis of Salt Iodine Content: Laboratory Performance, User- and Field-Friendliness

**DOI:** 10.1371/journal.pone.0138530

**Published:** 2015-09-24

**Authors:** Fabian Rohner, Marcelline O. Kangambèga, Noor Khan, Robert Kargougou, Denis Garnier, Ibrahima Sanou, Bertine D. Ouaro, Nicolai Petry, James P. Wirth, Pieter Jooste

**Affiliations:** 1 GroundWork LLC, Crans-près-Céligny, Switzerland; 2 Laboratoire National de Santé Publique, Ouagadougou, Burkina Faso; 3 The Micronutrient Initiative, Ottawa, Canada; 4 UNICEF, Ouagadougou, Burkina Faso; 5 Direction de la Nutrition, Ministère de Santé, Ouagadougou, Burkina Faso; 6 Iodine Global Network South Africa, Cape Town, South Africa; 7 Centre of Excellence for Nutrition, North-West University, Potchefstroom, South Africa; Rutgers University, UNITED STATES

## Abstract

**Background:**

Iodine deficiency has important health and development consequences and the introduction of iodized salt as national programs has been a great public health success in the past decades. To render national salt iodization programs sustainable and ensure adequate iodization levels, simple methods to quantitatively assess whether salt is adequately iodized are required. Several methods claim to be simple and reliable, and are available on the market or are in development.

**Objective:**

This work has validated the currently available quantitative rapid test kits (quantRTK) in a comparative manner for both their laboratory performance and ease of use in field settings.

**Methods:**

Laboratory performance parameters (linearity, detection and quantification limit, intra- and inter-assay imprecision) were conducted on 5 quantRTK. We assessed inter-operator imprecision using salt of different quality along with the comparison of 59 salt samples from across the globe; measurements were made both in a laboratory and a field setting by technicians and non-technicians. Results from the quantRTK were compared against iodometric titration for validity. An ‘ease-of-use’ rating system was developed to identify the most suitable quantRTK for a given task.

**Results:**

Most of the devices showed acceptable laboratory performance, but for some of the devices, use by non-technicians revealed poorer performance when working in a routine manner. Of the quantRTK tested, the iCheck^®^ and I-Reader^®^ showed most consistent performance and ease of use, and a newly developed paper-based method (saltPAD) holds promise if further developed.

**Conclusions:**

User- and field-friendly devices are now available and the most appropriate quantRTK can be selected depending on the number of samples and the budget available.

## Introduction

It is widely accepted that iodine deficiency, even in its milder forms, has important consequences on mental development, intellectual capacity and growth [1]. Salt iodization is a highly cost-effective approach to combating iodine deficiency [2], and universal salt iodization programs in many countries have increased the coverage of iodized table salt over the past two decades, and thereby, reduced iodine deficiency and its disorders. In 1993, over 110 countries were estimated to be affected by any form of iodine deficiency; this number was reduced to 54 in 2003 and 30 in 2013 [3]. Simultaneously, a number of countries with excessive iodine intakes have emerged, indicating that it is important to keep a fine balance when iodizing salt; iodine should be added to salt in the correct amounts and not in excessive quantities in order to prevent disorders such as iodine induced hyperthyroidism and thyrotoxicosis. Therefore, the assessment of the adequacy of salt iodization has gained importance and attention, since it is no longer sufficient to just verify whether there is iodine in the salt or not but rather, how much iodine it contains. This growing need to assess adequacy undermines the usefulness of qualitative (or at best, semi-quantitative [4]) rapid test kits that have been used extensively and for which a wide range of products exist [5].

Analytical methods quantitatively assessing iodine content in salt are required to carefully monitor the adequacy of salt iodization at the level of production, importation and consumption. While for most settings, iodometric titration has been accepted as the reference method, it requires a laboratory setting and equipment, as well as skilled personnel to handle the methodology correctly [6]. A series of apparently more user- and field-friendly analytical devices for the quantitative assessment of salt iodine content have become available in the recent past (hereafter referred to as ‘quantitative rapid test kits’, quantRTK). For some devices, methodological validation reports have been published [7–9], whilst for others, only unpublished reports exist. Overall though, there has thus far not been a comparative validation of these devices alongside each other.

This work was therefore conducted to compare the accuracy, precision, robustness and field-friendliness of the currently available quantRTK both under laboratory and field conditions and “real life scenario”. In addition to this objective assessment, a more subjective appraisal of logistical parameters, such as cost, availability and reliability of quantRTK and reagents, use of hazardous reagents, and disposal of used material was included in this work.

The overarching goal of the validation was to provide evidence-based guidance to professional staff from NGO’s, the UN, and governments and industry on the appropriate choice of an iodine measuring device for their specific use, including a discussion of flaws in the currently available systems and suggestions on how to overcome them in future developments.

## Material and Methods

This validation only used salt samples iodized with potassium iodate (KIO_3_); although most of the quantRTK examined can measure potassium iodide (KI), KIO_3_ is used considerably more often than KI for the iodization of table salt. For those methods that were not specifically developed for direct measurement of KI, an oxidation to KIO_3_ prior to analysis would be required using bromine water and formic acid [10].

### Description of the devices

For each of the quantRTK, a short description of the methodology used and the testing system is provided in Table 1. The names used in this description (mostly trade names) will be used thereafter in the manuscript. The measurement procedures followed for each quantRTK are described further below.

**Table 1:** Overview and short description of (electronic) rapid test kits used in this validation.

Device name	Manufacturer	Method principle	Description of test kit contents
iCheck iodine(‘iCheck’)	BioAnalyt LLC, Potsdam, Germany (www.bioanalyt.com)	Reduction of iodate to iodine by potassium iodide, followed by the formation of penta-iodide anions that inside the helical β-amylose chain of starch form a blue color that is linear with the iodine concentration. Colorimetric quantification of the concentration using photospectrometry.	Device, scale and power plug come with the device kit; activation solution, reagent vials, syringes and needles come with the reagent kits. Only purified water and plastic flasks required.
ID-ERTK (‘ID-ERTK’)	Innovative Design, Chennai, India (currently, no website available)		Device comes with power plug, scale, quartz cuvette, scale and some other lab hardware.Reagent solutions need to be prepared by the user.
I-Reader(‘I-Reader’)	Mahidol University, Institute for Innovative Learning, Mahidol, Thailand (http://www.il.mahidol.ac.th/eng/index.php/2012-09-11-07-32-25/test-kits/31-new-quantitative-method-for-determination-of-iodate-in-salt.html)		Device comes with pyrex tubes and tube holder, disposable pipettes and small dosage spoons (to measure the salt volumetrically rather than by weight), and a basic reagent stock (two bottles, sufficient for approx. 330 analyses). Scale not included.
saltPAD(‘saltPAD’)	University of Notre Dame, Notre Dame, Indiana, USA (http://padproject.nd.edu/)	Iodate is reduced to triiodide using potassium iodide. Thiosulfate is used to titrate a predetermined amount of triiodide. Excess triiodide reacts with starch to form a blue color that can be calibrated for visual or computerized image analysis.	This device is not yet commercially available, beta-testing version was used for this evaluation. Besides the testing cards that were delivered by the developer, a light box had to be constructed to take pictures for automated analysis, as well as a standard calibration series had to be prepared for calibration of the software to the specific light conditions.
WYD iodine checker (‘WYD’)	Salt Research Institute of China, National Salt Industry Corporation, Tianjin, PR China (website in Chinese only)	Same as iCheck, ID-ERTK and I-Reader.	Test kit comes with device and power plug, some lab hardware and a manual. Scale not included. Reagent solutions need to be prepared by the user.
MBI-RTK*(‘RTK’)	MBI Kits International, Chennai, India (www.mbikits.com)	Same principle, but no ‘quantitative’ instrumentation.	Comes with check solution, re-check solution and color-scale. No other material required.

*This test is semi-quantitative or qualitative only and was only included for the analysis of the blinded samples to be able to calculate the kappa-statistics.

### Description of validation steps

**Fig 1:**
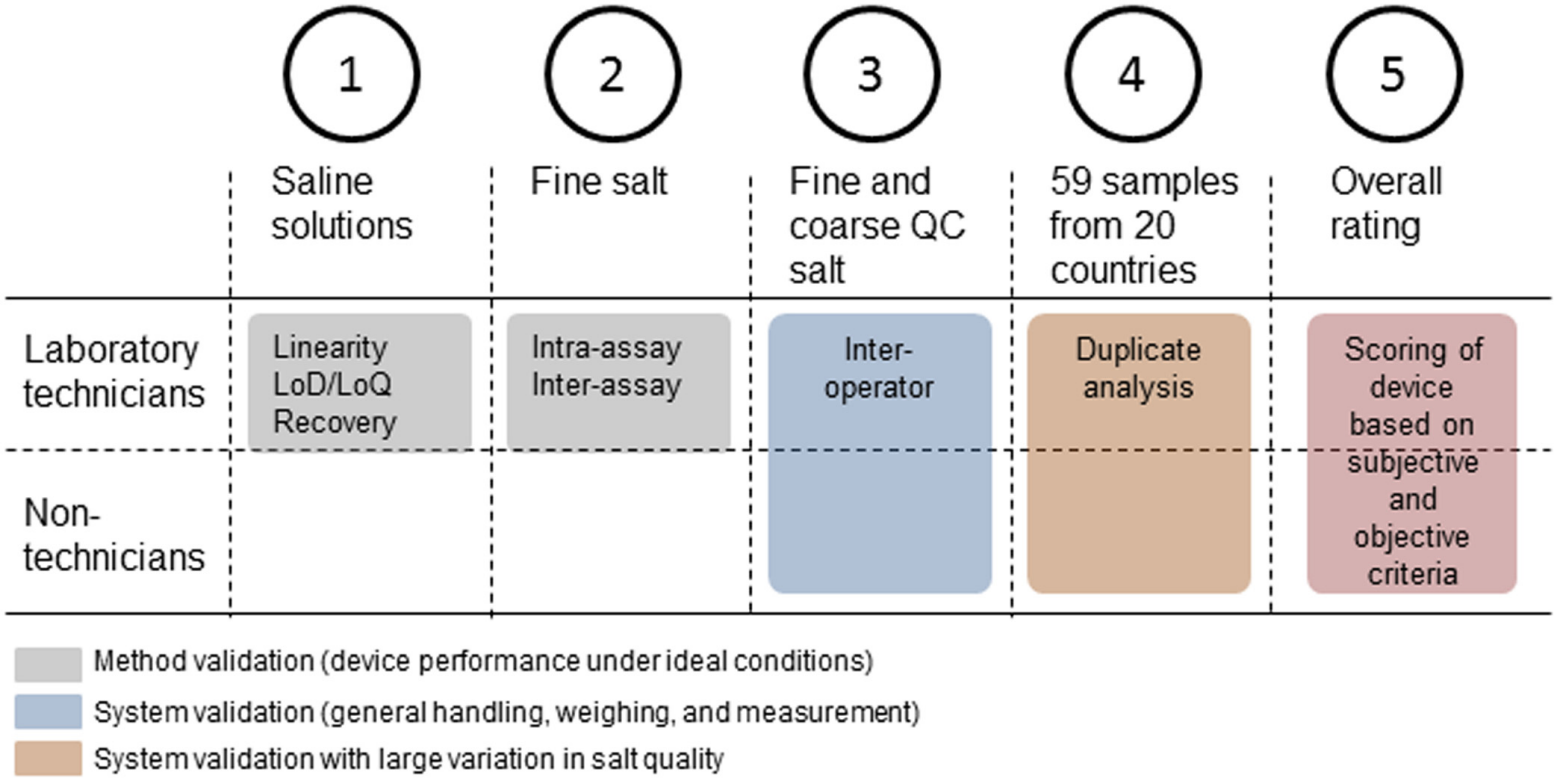
Overview of the various validation steps conducted on the different quantRTK’s.

As stipulated above, this work did not solely intend to assess the analytical performance of the quantRTK’s under ideal conditions, i.e. in a laboratory setting, being manipulated by highly trained technicians and using saline solutions with varying KIO_3_ concentrations; but also, it aimed at assessing the device-sample-operator system as a whole, since this is of most relevance when assessing the user- and field friendliness of such a device. In order to capture this part as well, several validation steps were conducted using different matrices and laboratory settings. An overview of the stages is provided in Fig 1.

Below, we present these validation steps conducted to assess the usefulness of the portable device. Where saline solutions were used, iodine content in mg/L has been converted to mg I/kg of salt (mg/kg). More details on the salt samples used are provided further below.

**Fig 2:**
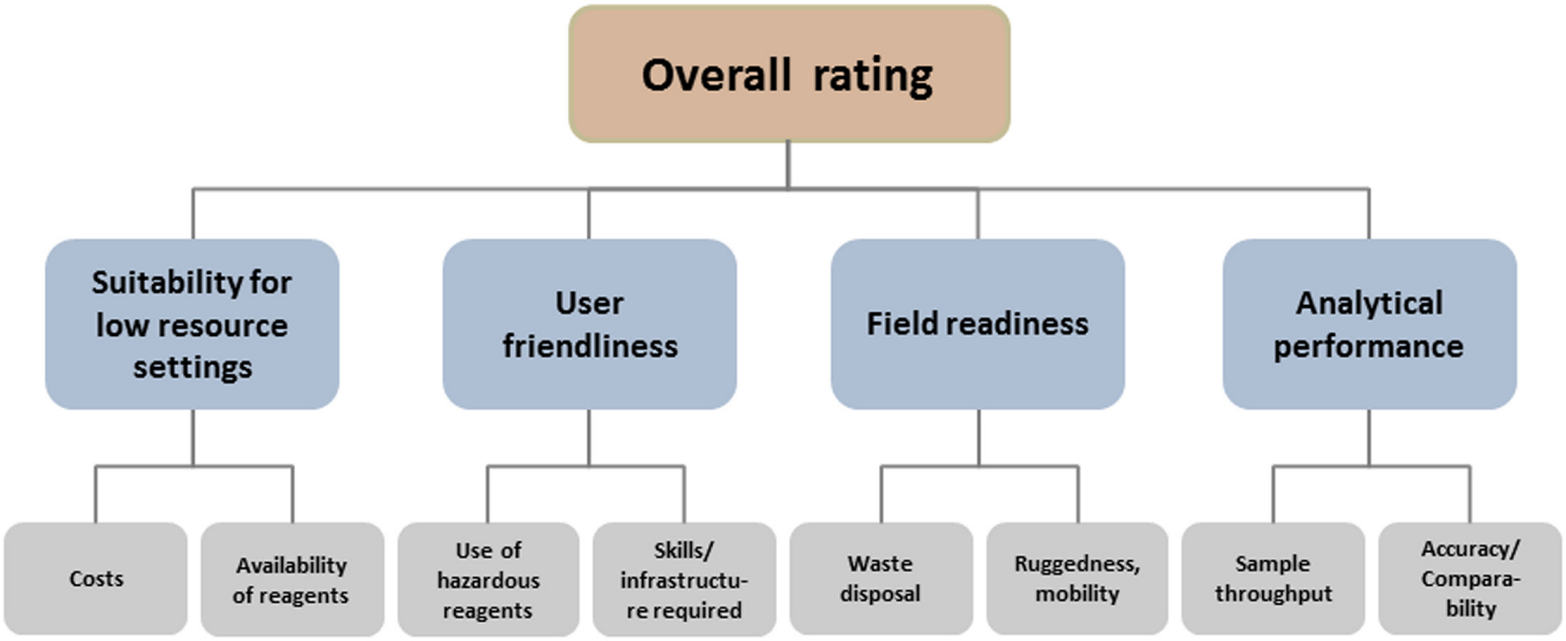
Schematic presentation of rating matrix employed.

1. **Using saline solutions.** Linearity of the portable device was determined by measuring in duplicate seven standard solutions (KIO_3_ in 20% w/w NaCl-solution) with iodate concentrations of 0, 3.0, 6.0, 9.0, 12.0, 15.0, 18.0 and 21.0 mg/L iodine as KIO_3_; depending on the dilution factor used for each device, this corresponds to a iodine content of 0 to ≈ 100–105 mg/kg for most of the devices.
  Limits of detection and determination: NaCl *puriss*. *p*. *a*. (Sigma-Aldrich, 31434) was dissolved in purified water (20% w/w), and the solution was measured ten times. Limit of detection: mean + 3 SD of the measurements; limit of determination: mean + 10 SD of the measurements [11].
  Method recovery (recovery A): the standard solutions (KIO_3_ in NaCl) prepared for the linearity assessment were used to calculate recovery; for this, the results falling within the measurement range were compared to the expected concentration (expected/observed *100).
2. **Using high quality, fine salt.** Intra-assay imprecision was assessed by preparing a solution of three high quality salt samples of varying but known iodine concentration (15.0 mg/kg, 29.6 mg/kg, and 59.1 mg/kg) and measuring them in 10 replicates; the coefficient of variation (CV) for each level was calculated.
  Inter-assay imprecision was determined by one technician conducting five analyses of each of three same salt samples over 3 days; the coefficient of variation (CV) for each level was calculated.
3. **Using both fine and coarse salt.** Inter-operator imprecision: three technicians measured the solutions of three salt samples of varying concentrations (15.0 mg/kg, 29.6 mg/kg, and 59.1 mg/kg) in five replicates of fine salt on the same day; the same was repeated using coarse salt with iodine contents of 20.0, 47.5, and 90.4 mg/kg, and the two inter-operator exercises were also conducted by three non-technicians in a field laboratory setting; for each salt type and laboratory setting the coefficient of variation (CV) for each level was calculated.
  System recovery (recovery B): Using the results from the inter-operator exercise, recoveries for the three levels of fine and coarse salt were calculated for the three technicians.
4. **Using a wide range of salt samples.** Comparison of the quantRTKs to the reference method: the concentration of iodine was measured in 59 salt samples from different countries of origin (using KIO3 as fortificant) with the quantRTKs, and results were compared to iodometric titration. For this, each sample was analysed in duplicate measurements. In order to include the qualitative rapid test kits that are frequently used in Demographic Health Surveys and Multiple Indicator Cluster Surveys, these 59 samples were also measured using the qualitative rapid test kits.
5. **Overall assessment.** To provide the reader an assessment not only of laboratory parameters, but also with information on the user- and field-friendliness, including cost, availability, handling of hazardous material and waste management, a rating system was established with scores ranging from 0 (lowest) to 5 (best) according to the categories presented in Fig 2. Because it was considered that analytical performance is of foremost importance, its score was multiplied by two.

### Description of salt samples used

For the validation steps 2 and 3, a high quality, fine vacuum non-iodized salt was iodized (using KIO_3_) close to pre-set levels of 15, 30, and 60 mg/kg, and these levels were confirmed using iodometric titration as 15.0, 29.6, and 59.1 mg/kg. These samples were subsequently used for the intra-assay, as well as for the inter-day and -operator imprecision. Additionally for step 3, to more thoroughly test inter-operator precision of the quantRTK, lower quality coarse salt from Senegal containing different levels of KIO_3_ was used. By titration, the levels of the 3 coarse salts were 20.0, 47.5, and 90.4 mg/kg.

For the validation step 4, 60 commercially available salt samples of varying iodine content (as KIO_3_; range 0 to 136 mg/kg), of different granularity (fine, medium, coarse) and different quality (from clean and dry samples to those containing foreign matter and moist) were obtained from 20 countries (Africa 7, South America 2, Asia 9, Europe 2). The reasoning behind such a wide variety of salt origin was to ensure that the testing results will be recognized by a wide range of country stakeholders.

### Measurement procedures followed

For each of the devices, the instructions provided by the supplier were followed as closely as possible, with a few exceptions (see S1 File):

- For the iCheck, the ID-ERTK, and the I-Reader, results that were above the measuring range (≈65 mg/kg for the iCheck, 50 mg/kg for the ID-ERTK, and 95 mg/kg for the I-Reader) were not diluted as per the supplier’s recommendation in order to demonstrate the measurement range.
- For the ID-ERTK, because the pipettes supplied with the kit were very difficult to handle by the technicians, adjustable volumetric pipettes were used instead.
- The I-Reader’s instructions propose to weigh 0.1 g of salt to be dissolved in 0.5 mL of distilled water. Because there were also coarse samples to be analysed leading to increased heterogeneity, the supplier was contacted to discuss an increase to 1 g of salt and 5 mL of water, which the supplier agreed to; this included an intermediate transfer step, for which adjustable volumetric pipettes were used.
- For the evaluation of linearity, limit of detection/quantification and intra-assay of the saltPAD, adjustable volumetric pipettes were used instead of the disposable pipettes in order to minimize bias during instrument evaluation; for this part of the validation, the results as interpreted by an expert reader were used. For the method validation (device plus handling) though, disposable pipettes were used to mimic field application. For the method validation, the operator’s readings and the software’s readings were used, since these two best mimic routine use.
- The instructions of the WYD suggest using diluting different amounts of salt depending if the salt is fine or coarse. In this study, 1 g was weighed regardless of whether it was fine or coarse salt, since such differing instructions would lead to confusion during the preparation of samples.

The reagents and quantRTK’s were stored at room temperature both at the laboratory and the field lab setting, which means that storage conditions were dry but rather warm (28–38°C). The salt quantities indicated by the manuals were weighed using portable scales (various brands, as provided by the suppliers of the quantRTK’s) with a precision of ±0.01g and diluted in the appropriate quantities (by weight, except for the WYD) of ultrapure water (ELGA, Purelab Classic).

### Reference method

The reference method, iodometric titration, was conducted according to AOAC International [12], with slight modifications by the ‘Laboratoire National de Santé Publique’ in Burkina Faso, which undertakes regular external quality control. Given the importance of the accuracy of the reference method, samples for the second part of the validation (analysis of the 60 samples) were analysed by one of the quality control laboratories of Suedsalz GmbH (Bad Reichenhall, Germany), a private company laboratory that regularly takes place in external quality assurance programs. This laboratory conducts iodometric titration according to EuSalt analytical standards [13] on a fully automated platform. The results on the reference method from the two laboratories were in excellent agreement (y = 0.94x+4.1, R^2^ = 0.98; except for one sample which was excluded when after several repetitions the discrepancy persisted). For the included sample results, the average of the two laboratories was used to establish the reference value (n = 59).

### Data analysis

For the laboratory validation of the method, standard protocols were followed, unless otherwise described. Data processing and statistics were conducted using Microsoft Excel 2010. For the method comparison, besides plotting the two data sets and calculating the Spearman coefficient and the regression equation, the Bland-Altman plot was used [14].

Limits of agreement (LOA) were calculated using

$${LOA}_{low}\;=\;\Delta -2SD$$

$$LOA_{high}\;=\;\Delta +2SD,$$

where Δ is the mean of the difference between two methods.

For the inclusion of the RTK in the method comparison, quantitative results were dichotomized into iodine concentrations <15 mg/kg and ≥15 mg/kg and kappa values, sensitivity, specificity, positive and negative predictive values were calculated for qualitative inter-rater agreement [15,16].

## Results

Table 2 shows the main findings of the assessment of the analytical performance of the evaluated quantRTK’s, when minimizing the ‘system’ interference, e.g. by having only one technician conducting the tests and by using either KIO_3_ solutions in NaCl solution of high purity or high quality salt.

**Table 2:** Summary of results from the ‘method’ validation of the different quantRTK’s.

Device	Measuring range	Linearity (R^2^)	LoD/ LoQ (mg/kg)^a^	Intra-assay imprecision (% CV)^b^	Recovery A (%)^c^	Recovery B (%)^c^	Inter-assay imprecision (% CV)^b^
**iCheck**	< ≈65 mg/kg^d^	0.9877	5.7/ 6.3	1.9, 3.9, 4.9	103 ± 11	99 ± 17	4.5, 3.8, 6.6
**ID-ERTK**	15–50 mg/kg^e^	0.9181	n/d^e^	n/d, 8.2, 13.3^e^	80 ± 12	79 ± 26	n/d, 8.5, 5.7
**I-Reader**	< ≈90 mg/kg	0.9984	0.0/ 0.0	5.0, 2.6, 5.3	92 ± 2	110 ± 31	5.0, 7.1, 5.9
**saltPAD** ^**f**^	< ≈65 mg/kg	0.8966	0.0/ 0.0	0.0, 37.0, 15.0	97 ± 22	80 ± 14	19.4, 39.4, 30.9
**WYD**	< ≈95 mg/kg	0.9974	0.0/ 0.0	4.6, 1.4, 1.4	90 ± 4	70 ± 17	7.6, 5.4, 7.6

^a^ LoD, limit of detection; LoQ, limit of quantification; for the description of the calculations, refer to the description in the method section;

^b^ three iodine levels were used (15.0, 29.6, 59.1 mg/kg) and the three CV’s are given in the order of increasing iodine concentration;

^c^ Recovery A was calculated from the linearity assessment, and results are presented as mean recovery ± SD; Recovery B was calculated from the inter-operator precision exercise and comprises the observed/expected values from the samples with approximate KIO_3_ concentrations of 15, 20, 30, 45, 60, and 90 mg/kg; results are shown with % SD.

^d^ the device gives results in mg/L and anything above 13mg/L is indicated as 'above measuring range'; assuming 1:5 dilution (factor 5.45), this corresponds to 65 mg/kg;

^e^ The device has set working ranges from 15–50 mg/kg and thus, LoD and LoQ could not be assessed; further, for intra- and inter-assay imprecision and recovery, the low level of salt (15.0 mg/kg) could not be assessed; the high level yielded results, because the device gave consistently lower readings; n/d thus, means not determined;

^f^ For the saltPAD, three types of interpretation of the results on the cards were done: interpretation by the operator, by an expert reader (a person from the device developer) and an image analysis software; for the device performance, the expert reader’s results only were used.

### Measuring range

The measuring range is presented as approximations only, since the different dilution factors make it difficult to exactly define the lower and upper range. While for the WYD and the I-Reader, the measuring range hardly posed a problem in the samples analysed, for the iCheck, the measuring range is such that higher values could not be measured (in our case, these were three samples of ≈80 and ≈140 mg/kg, including the high level for the inter-operator imprecision of coarse salt) and for ID-ERTK, which has a set upper limit but also a lower limit, which resulted in many missing data points in our validation. For the saltPAD, the cards are conceptualized such that up to ≈50 mg/kg, the results can be read in 5 mg/kg-steps, followed by 10 mg/kg increments up to 75 mg/kg; for the next level, the cards only provide a range of 75–150 or > 150 mg/kg. Thus, because of the different concept, it is difficult to set a measuring range, since the range is large but with decreasing resolution; here, we indicate the range for which a linearity regression could be calculated.

### Linearity, detection and quantification limit

Most methods showed a linear behaviour in the measuring range of the device (iCheck, ID-ERTK) or up to the evaluated range (0 to ≈100 mg/kg); for the ID-ERTK, there was a non-linear trend at the higher end of the measuring range. The assessment of LoD and LoQ proved to be a challenge in this validation, since two of the devices gave consistently ‘0’ values on the screen (WYD, I-Reader), which seems not realistic for such methods, but we could not find out from the device manufacturers if there is a built-in algorithm to set low readings to zero. For the ID-ERTK, these limits could not be assessed due to the built-in range limit. For the iCheck device, the LoD and LoQ are 5.7 and 6.3 mg/kg, which is slightly above what would be considered a threshold for non-iodized salt (> 5 mg/kg; [17]). The LoD and LoQ of the saltPAD are both 0, because the expert reader interpreted all corresponding cards as having no iodine.

### Recovery

To demonstrate recovery, two approaches are used:

- Recovery A: the linearity tests were used to calculate recovery from known amounts of KIO_3_, knowing that this is a recovery under ideal conditions (no impurities, no inhomogeneity, etc.). For the iCheck, and the saltPAD, recoveries are within 5% of the expected concentration over the measuring range, whereas for the I-Reader and WYD, a slightly lower recovery was found, but still within 10% from the expectations. The recovery of the ID-ERTK was just over 80% compared to the expected concentrations.
- Recovery B: results from the inter-operator assessment were used to calculate recoveries, assuming that this type of recovery includes variability originating from the salt sample type and the operator. As expected, these recoveries show a wider variation, but the iCheck and I-Reader are still within 10% of the expected values. In contrast, the three other devices tend to underestimate iodine concentrations in salt samples.

### Intra- and inter-assay imprecision

With the exception of the ID-ERTK and saltPAD, intra- and inter-assay imprecision was below 10% for the remaining devices, in most of the cases even around or below 5%. By far the largest intra- and inter-assay bias was observed for the saltPAD, which is not surprising when considering the concept of the 5 mg/kg steps rather than a continuous measure. This is of particular relevance in the lower range, where 5 mg/kg off-readings can dramatically inflate the CV.

### Inter-operator imprecision

For the inter-operator imprecision, two teams of three evaluators were trained: one team of three technicians working in a laboratory environment and a team of non-technicians working in a field laboratory. Further, in addition to high-quality salt, lower quality coarse salt was included in this exercise. The results are presented in Table 3. While there is no consistent pattern of increasing imprecision from technicians to non-technicians, there is clearly an increased imprecision for the WYD, I-Reader and saltPAD when it comes to coarse salt; this can certainly in part be explained by the smaller amounts of salt weighed in for the analysis (1 g for WYD and I-Reader, 3.25 g for saltPAD) compared to the other devices (10 g) and thus, an increased heterogeneity coming from the salt.

**Table 3:** Summary of the results from the ‘system’ validation: inter-operator imprecision, expressed as coefficient of variation.

	Technicians		Non-technicians	
Device	Fine salt (%CV)^a^	Coarse salt (%CV)^b^	Fine salt (%CV)^a^	Coarse salt (%CV)^b^
**iCheck**	13.3, 6.8, 9.7	10.1, 4.0, n/d^c^	4.2, 5.2, 4.3	7.8, 3.0, n/d^c^
**ID-ERTK**	n/d^c^, 7.8, 12.5	n/d^c^, 6.3, n/d^c^	n/d^c^, 11.7, 7.7	n/d^c^, 4.7, n/d^c^
**I-Reader**	7.2, 12.5, 5.1	26.7, 11.6, 9.1	9.4, 11.1, 8.4	28.7, 23.8, 18.4
**saltPAD**_**operator**_ ^**d**^	18.6, 35.7, 23.1	21.3, 17.7, 21.5	5.9, 19.4, 21.7	15.3, 28.9, 10.3
**saltPAD**_**software**_ ^**d**^	32.7, 17.6, 23.9	32.9, 38.6, n/d^c^	23.6, 9.5, n/d^c^	28.1, 19.0, n/d^c^
**WYD**	7.2, 12.1, 5.1	26.7, 11.6, 9.1	18.8, 7.5, 5.3	13.5, 6.7, 7.6

^a^ three iodine levels were used (15.0, 29.6, 59.1 mg/kg) and the three CV’s are given in the order of increasing iodine concentration;

^b^ three iodine levels were used (20.0, 47.5, 90.4 mg/kg) and the three CV’s are given in the order of increasing iodine concentration;

^c^ outside of measuring range for more than one measurement and thus, n/d means not determined.

^d^ For the saltPAD, three types of interpretation of the results on the cards were done: interpretation by the operator, by an expert reader (a person from the device developer) and an image analysis software; the index provides the information which readings were used.

For the saltPAD, there is the additional factor of inter-operator variation in the interpretation of the cards. But even when relying on the expert readings, the respective imprecision estimates are 20.6, 24.4, and 18.1% for the fine salt done by technicians; 17.8, 21.0%, and n/a (over 75 mg/kg) for the fine salt done by non-technicians; 16.0, 35.1, and 9.7% for the coarse salt done by technicians; 27.7, 24.9%, and n/a (over 75mg/kg) for the coarse salt done by non-technicians.

### Comparison to reference method

**Fig 3:**
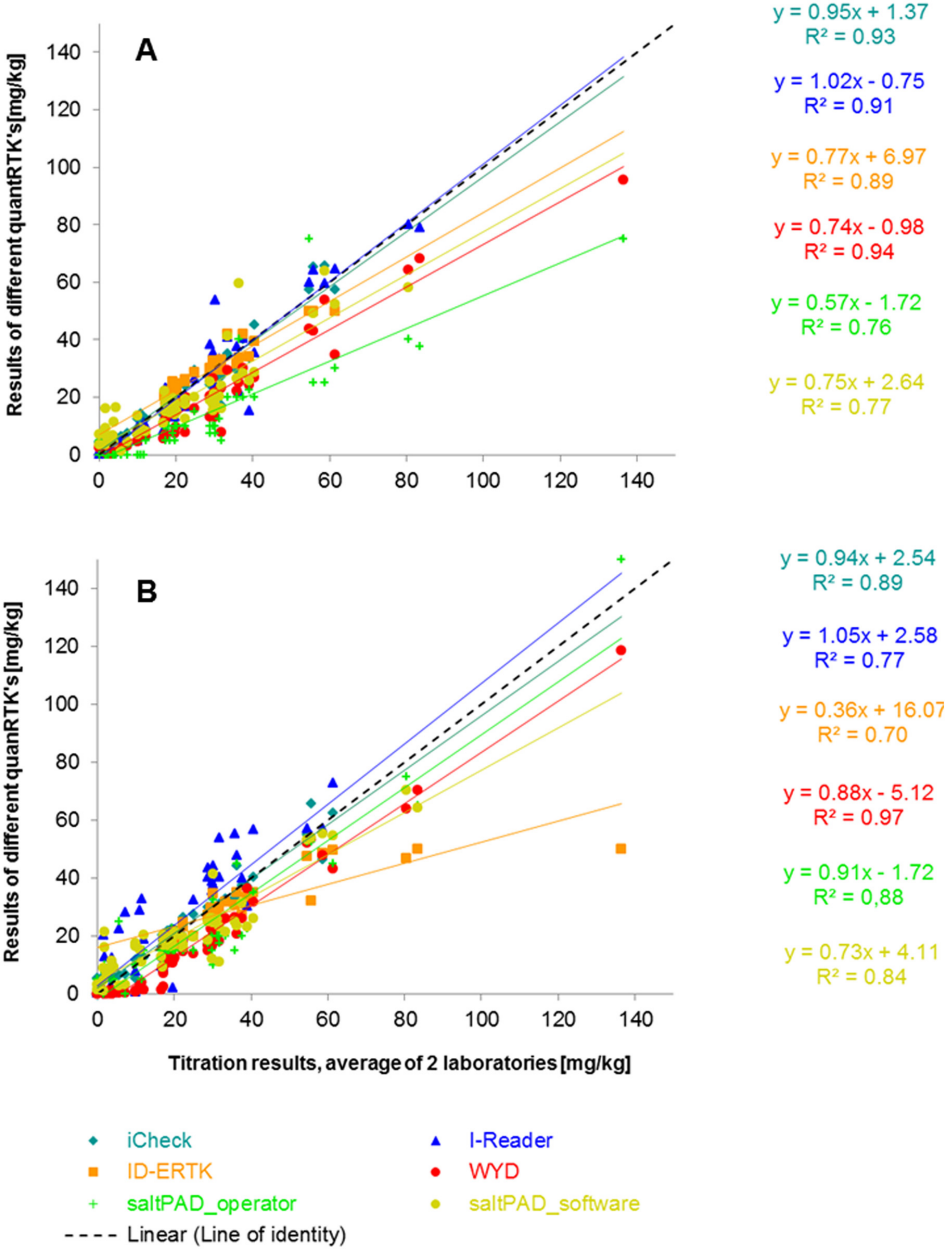
Regression plot of the comparison between the reference method and each quantRTK; A: technician’s analyses; B: non-technician’s analysis.

To further the system validation, 59 salt samples of various origins were measured in duplicates on each quantRTK, once conducted by a technician and once by a non-technician. The agreement between the reference method and each of the quantRTK is graphically presented once when conducted by a technician and once when conducted by a non-technician, see Fig 3A and 3B.

While the spearman coefficient was over 0.9 for the iCheck, I-Reader and the WYD, when manipulated by the technician, only the WYD was above this value for the non-technician, with the iCheck being close to 0.9. The ID-ERTK reaches a spearman coefficient close to 0.9 as well for the technician but performed poorly when used by the non-technician. The opposite is the case for the saltPAD, when the operators’ readings are used; however, when the software’s readings are used, the results from the non-technician and the technician are more comparable. Regarding the slope of the regression line, the iCheck and the I-Reader were close to 1 for both operators, but varied considerably for the other three devices.

Due to the wealth of comparisons, a graphic representation of the Bland-Altman plot proved to be difficult. Table 4 thus presents the key variables of the Bland-Altman concept, with Δ being the difference between the reference method and the quantRTK in question and the LOA’s. The WYD, iCheck and I-Reader gave a relatively narrow window for the LOA for both the technician and the non-technician (albeit less for the I-Reader), yet the WYD showed a positive offset for both operators. The iCheck’s window is narrowest for both operators with a minimal offset, followed by the I-Reader, still with a minimal offset but a slightly larger LOA window. For the ID-ERTK, only over half of the samples could be included in this continuous analysis (the remainder being outside of the measuring range), and the LOA window varies massively between the technician and the non-technician, as well as the offset from zero varies a bit. A similar pattern is observed for the saltPAD, but interestingly, the non-technician did considerably better. As previously mentioned, a factor interfering on this test is the interpretation and it appears yet again that there is a large inter-operator difference in interpretation, which is likely more important than test-handling itself. To corroborate this, the results as interpreted by analytical software show more consistency in the results between the technician and non-technician.

**Table 4:** Bland-Altman’s Limits of Agreement (LOA).

	Technician (mg/kg)				Non-technician (mg/kg)			
Device	n valid^a^	Δ^b^	LOA_low_ ^c^	LOA_high_ ^c^	n valid^a^	Δ^b^	LOA_low_ ^c^	LOA_high_ ^c^
**iCheck**	56	-0.4	-8.8	8.0	56	-0.9	-8.3	6.5
**ID-ERTK**	33	-0.2	-8.1	7.7	34	7.9	-24.9	40.8
**I-Reader**	58	0.3	-11.7	12.3	56	-3.5	-21.2	14.2
**saltPAD**_**operator**_ ^**d**^	59	12.5	-13.0	38.0	59	4.0	-12.6	20.0
**saltPAD**_**software**_ ^**d**^	56	2.8	-13.3	18.8	58	2.1	-13.8	18.0
**WYD**	59	7.5	-7.9	22.9	59	8.0	-1.3	17.3

^a^ Provides the number of samples with a valid quantitative result (i.e. not below or above the measuring range);

^b^ Difference between the reference method and the respective quantRTK;

^c^ Difference between the reference method and the quantRTK ±2 SD.

^d^ For the saltPAD, three types of interpretation of the results on the cards were done: interpretation by the operator, by an expert reader (a person from the device developer) and an image analysis software; the index provides the information which readings were used.

In order to be able to take into consideration all available samples and to also include the qualitative MBI-RTK which has been widely used globally, the results were dichotomized into non-/inadequately iodized (<15 mg/kg) and adequately iodized salt (≥ 15 mg/kg) and the rating agreement between the reference method and the respective quantRTK (including the MBI-RTK) using the Kappa value, sensitivity, specificity and positive and negative predictive values are presented in Table 5. These results, albeit ignoring a wealth of information coming from the continuous results, show that quantitative devices more resembling laboratory devices (including pipetting and reading results of a screen) do better when manipulated by technicians than non-technicians; further, in this validation, the qualitative RTK performed poorest, even on dichotomized data.

**Table 5:** Kappa-value, sensitivity, specificity, positive and negative predictive values for the qualitative method comparison of titration and quantRTK/ RTK (n = 59).

	Parameter	iCheck	ID-ERTK	I-Reader	saltPAD_operator_ ^a^	saltPAD_software_ ^a^	WYD	RTK
**Technician**	**Kappa-value,—(95% CI)**	0.89 (0.77, 1.00)	0.85 (0.71, 0.99)	0.93 (0.82, 1.00)	0.41 (0.25, 0.58)	0.74 (0.56, 0.92)	0.63 (0.45, 0.81)	0.09 (-0.11, 0.28)
	**Sensitivity, % (95%CI)**	92.5 (78.5, 98.0)	92.5 (78.5, 98.0)	95.0 (81.8, 99.1)	45.0 (29.6, 61.3)	87.5 (72.4, 95.3)	72.5 (55.9, 84.9)	37.5 (23.2, 54.2)
	**Specificity, % (95%CI)**	100.0 (79.1, 100.0)	94.7 (71.9, 99.7)	100.0 (79.1, 100.0)	100.0 (79.1, 100.0)	89.5 (65.5, 98.2)	100.0 (79.1, 100.0)	73.7 (48.6, 89.9)
	**PPV, % (95%CI)** ^**b**^	100.0 (88.3, 100.0)	97.4 (84.6, 99.9)	64.4 (50.8, 76.1)	100.0 (78.1, 100.0)	94.6 (80.5, 99.1)	100.0 (85.4, 100.0)	75.0 (50.6, 90.4)
	**NPV, % (95%CI)** ^**c**^	86.4 (64.0, 96.4)	85.7 (62.6, 96.2)	90.5 (68.2, 98.3)	46.3 (31.0, 62.4)	77.3 (54.2, 91.3)	63.3 (43.9, 79.5)	35.9 (21.7, 52.8)
**Non-technician**	**Kappa-value,—(95% CI)**	0.89 (0.77, 1.00)	0.75 (0.57, 0.92)	0.60 (0.38, 0.82)	0.80 (0.64, 0.97)	0.60 (0.38, 0.82)	0.52 (0.33, 0.70)	0.44 (0.24, 0.64)
	**Sensitivity, % (95%CI)**	92.5 (78.5, 98.0)	85.0 (69.5, 93.8)	90.0 (75.4, 96.7)	95.0 (81.8, 99.1)	90.0 (75.4, 96.7)	62.5 (45.8, 76.8)	62.5 (45.8, 76.8)
	**Specificity, % (95%CI)**	100.0 (79.1, 100.0)	94.7 (71.9, 99.7)	68.4 (43.5, 86.4)	84.2 (59.5, 95.8)	68.4 (43.5, 86.4)	100.0 (79.1, 100.0)	89.5 (65.5, 98.2)
	**PPV, % (95%CI)**	100.0 (88.3, 100.0)	97.1 (83.4, 99.9)	85.7 (70.8, 94.1)	92.7 (79.0, 98.1)	85.7 (70.8, 94.1)	100.0 (83.4, 100.0)	92.6 (74.2, 98.7)
	**NPV, % (95%CI)**	86.4 (64.0, 96.4)	75.0 (52.9, 89.4)	76.5 (49.5, 92.2)	88.9 (63.9, 98.1)	76.5 (49.8, 92.2)	55.9 (38.1, 72.4)	53.1 (35.0, 70.5)

^a^ For the saltPAD, three types of interpretation of the results on the cards were done: interpretation by the operator, by an expert reader (a person from the device developer) and an image analysis software; the index provides the information which readings were used.

^b^ Positive predictive value;

^c^ Negative predictive value.

On the other hand, methods using a different concept do better under a less-trained operator, such as saltPAD and RTK. The exception to this is the iCheck, where both operators yielded very similar results. For the WYD, the apparent contradiction to Fig 3 is explained by the difference in the regression line: whilst for the technician the slope is below 1, for the non-technician there is a systematic offset but the slope is close to 1. The former plays a more important role at higher concentrations and therefore, when dichotomizing values using a low cut-off, these parameters are less affected. The ID-ERTK also shows a better performance on the dichotomized data, most likely because the device seems to adequately categorize despite a small quantitative measuring range. As previously, when using the results for the saltPAD as obtained through the software, the difference between the technician vs. non-technicians are much smaller.

## Discussion

Simple quantitative analysis of salt iodine content is increasingly important to not only state whether salt contains any iodine or not but to assess whether it contains adequate levels established by national governments or following international regulations (15–45 mg/kg;[3]. Several quantRTK are now available, either in a commercialized form or in development stage. This study aimed at evaluating such devices for their analytical performance but also for their field- and user-friendliness as well as their suitability for low resource settings.

To our knowledge, this is the first study that has evaluated currently available quantRTK in a comparative manner at the same time and under very similar conditions, and has compared the performance of each device in both a lab setting used by technicians and in a field setting used by non-technicians.

Along with the analytical performance results, ‘softer’ parameters were evaluated in as objective a manner as possible. These include cost and availability of the device and reagents, the use of hazardous reagents, skills and infrastructure required, waste generation, and device ruggedness and mobility (i.e. independence from electricity and computers). The analytical performance has been presented in the previous chapters, including the comparison of use by technicians and non-technicians, and the other parameters are shown in detail in S1 Table. To present the overall assessment in a condensed form, a rating system was created and the results are shown in Table 6 below.

**Table 6:** Overall assessment of the quantRTK included in the validation, including objective and subjective parameters.

Device name	Analytical performance	User friendliness	Field readiness	Suitability for low resource settings	Overall rating^a^
iCheck	4.5	3.7	4.3	3.3	**4.1**
ID-ERTK^b^	3.5	2.7	3.5	4.0	**3.4**
I-Reader	4.3	4.7	4.2	4.0	**4.3**
saltPAD^b^	3.5	4.0	4.2	3.0	**3.6**
WYD	3.8	2.7	3.7	3.7	**3.6**

^a^ Overall rating: (2*Analytical performance+user friendliness+field readiness+low resource setting suitability)/5;

^b^ These devices are not yet commercially available and under further development; thus, the scores are of transient nature.

In terms of analytical performance, user- and field-friendliness, the iCheck and the I-Reader have been rated consistently high, so that they score highest overall; analytical performance was scored highest due to the operator-independence. The iCheck has been rated relatively low in the suitability for low resource setting mainly due to its high upfront and per-sample cost. The I-Reader has the advantage over the iCheck that it gives results directly in mg/kg and no further conversion is required; this comes at the price of exact weighing required in order to maintain the validity of the results. One potential weakness of the I-Reader was observed during the validation: despite indicating normal functioning, the readings showed a sudden large shift although the same reagent bottle was in use. When changing to a new battery, this shift disappeared. This finding is something the developer should carefully look into, since this can be a challenge in a routine analysis setting.

In our validation, the WYD that was previously evaluated [7] did not rate highest in the analytical performance because of either a slope shift or a systematic offset in most measurements; because the shift is in the opposite direction as previously described, several tests were repeated using freshly prepared reagents and a different device with similar results. Also, given the need to prepare solutions using concentrated sulphuric acid and the fragility of some components (volumetric flasks and quartz cuvettes), the overall score is not excellent. This device can clearly be useful in a setting of central salt analysis, but whether its advantages outweigh those of titration is doubtful.

The ID-ERTK is currently only being commercialized in the Indian market; this explains the narrow measuring range of 15–50 mg/kg, which in our view is an important limitation of the device. Analytical performance assessment above indicates an acceptable performance in that narrow range, with a strong operator bias. This masks the fact that for this validation, we had to order 4 devices in total in order to have one functional one. The device’s production quality and finish is thus probably among the most important criticisms of this device; before commercializing it globally, the developer needs to improve the device’s ruggedness, increase its measuring range and carefully re-test all peripherals that were provided; for more details refer to S1 Table.

Though still under development, we got the opportunity to also include the saltPAD, a paper-based method using dry-chemicals, in our validation. Its inclusion required quite some improvisation, since the developer only sends the cards, instructions and a training manual for the visual readings of the results. If visual reading only is done, this is sufficient and only disposable pipettes are required. However, the visual readings turned out to be a current major bottleneck in the performance, since out of 6 personnel trained, 2 clearly mis-interpreted the results quite consistently, leading to poorer analytical performance. In order to avoid this, a ‘picture box’ with constant lighting and an opening to take digital images using a cell phone or tablet was constructed, and pictures sent to the developer for blinded expert’s readings and semi-automated image analysis using a software. This improved some of the analytical performance parameters albeit not all, but removed the large operator-bias, as it was mainly due to the visual interpretation. In order for the saltPAD to be usefulness in a routine setting, the developer will need to create a ‘kit’ with standardized light boxes, a suitable cell phone and ready-made calibration solutions. If this is successfully developed, the device may be useful for low-throughput, low-resource settings, e.g. in border outposts or alike.

The qualitative RTK was only included in a minor part of this validation but confirms what was found in previous evaluations, namely that there is operator-bias and that the qualitative between method-agreement is not excellent [4]. Due to its simple use, the RTK will likely continue to be used, but it should be carefully accompanied with quantitative analyses at critical points in order to obtain useful monitoring information.

## Conclusions

A range of devices to quantitatively analyse salt iodine content are now on the market or under development and of those included in this validation, two of them—the I-Reader and the iCheck—appear to be the most reliable in terms of analytical performance. The I-Reader is the more affordable option (both in terms of device cost and reagent cost) but requires minor modifications to be fully recommendable, including using larger salt quantities to address the heterogeneity issue of lower quality salt. The iCheck has higher cost (both in terms of device and reagent costs), currently requires a computer to calculate results and produces additional waste (e.g. needles and glass vials) but comes in a finished design otherwise and uses 10 g of salt. Two other devices, the WYD and the ID-ERTK have also performed acceptably but given the need to prepare solutions and thus, their requirement of basic laboratory setting and skills, their advantage over titration is not apparent. Additionally, the ID-ERTK has a very narrow measuring range; it has to be noted that this device is still in early stages of commercialisation. A device still under the development is the saltPAD, but in our view it is a promising approach if the inventors develop it into a ready-to-use kit with immediate and independent image analysis.

The user should nonetheless keep in mind that any device or method used to quantitatively assess the iodine content of salt should be accompanied by a well-functioning internal quality control system and participation in external quality assurance schemes. Both iodometric titration and quantRTK are subject to error due to inappropriate utilization, sudden instrument shift, or other problems, and quality control/assurance procedures can help ensure that measurement issues are identified in a timely manner.

## Supporting Information

S1 File Operator Instructions for the quantRTK. (PDF)

S2 File Dataset PlOS ONE. (XLSX)

S1 Table Supplementary table. (XLSX)

## Abbreviations

CV: coefficient of variation
LoA: limit of agreement
LoD: limit of detection
LoQ: limit of quantification
quantRTK: quantitative rapid test kit
