# Supplementary material for: Comparative Validation of Five Quantitative Rapid Test Kits for the Analysis of Salt Iodine Content: Laboratory Performance, User- and Field-Friendliness
Source: PLoS One. 2015 Sep 24;10(9):e0138530. doi: 10.1371/journal.pone.0138530 (PMC4581857; doi:10.1371/journal.pone.0138530)
Supplement: S1 File — (PDF) [file pone.0138530.s001.pdf]

This supplementary file contains the user-modified instructions in French that were used for the following study:

Comparative validation of five quantitative rapid test kits for the analysis of salt iodine content: laboratory performance, user- and field-friendliness

Fabian Rohner[1], Marcelline O. Kangambèga[2], Noor Khan[3], Robert Kargougou[4], Denis Garnier[4], Ibrahima Sanou[2], Bertine D. Ouaro[5], Nicolai Petry[1], James P. Wirth[1], and Pieter Jooste[6]

[1] GroundWork LLC, Crans-près-Céligny, Switzerland

[2] Laboratoire National de Santé Publique, Ouagadougou, Burkina Faso

[3] The Micronutrient Initiative, Ottawa, Canada

[4] UNICEF, Ouagadougou, Burkina Faso

[5] Direction de la Nutrition, Ministère de Santé, Ouagadougou, Burkina Faso

[6] Iodine Global Network South Africa, Cape Town, South Africa, Centre of Excellence for Nutrition, North-West University, Potchefstroom, South Africa

Please note that these instructions are not those endorsed by the suppliers of the different devices, nor are they comprehensive in terms of describing all steps involved but only those relevant for our study.

iCheck

1. Préparer l’échantillon

- Peser environ 10 g* de sel et enregistrer le poids exact dans votre documentation.
- Ensuite, mesurer ou peser dans environ 50 ml d'eau. Notez le poids exact.
- Mélanger votre échantillon de sel avec de l'eau jusqu'à ce sel est complètement solubilisé.

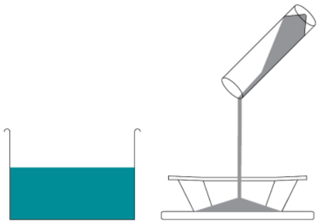

2. Activer les flacons de réactif Iodine

- Activer les flacons Iodine en injectant EXACTEMENT 0,2 ml (200 µl) de solution d‘additif (flacons à capuchon bleu) dans chaque flacon de réactif.
- Melanger lentement les solutions

→ Vous pouvez activer autant de flacon de réactif que vous utilisez dans 1 jour

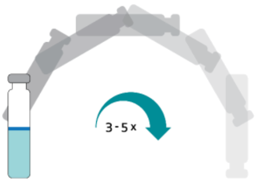

3. Injecter l’échantillon

- Injecter EXACTEMENT1 ml d’échantillon.
- Pour réduire la pression de l'air dans le flacon, faire sortir 1,0 ml d'air avant de retirer la seringue.
- Mélanger la solution en renversant lentement le flacon 3 à 5 fois et attendre 5 minutes.

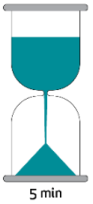

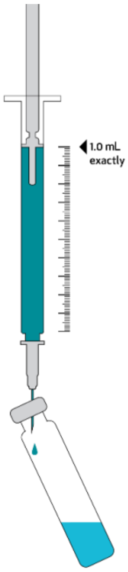

4. Insérer le flacon

- Ne pas toucher la partie transparente du flacon!
- Remettre le capuchon iCheck sur l’unité de mesure
- Allumer iCheck
- Appuyer sur flêche verte

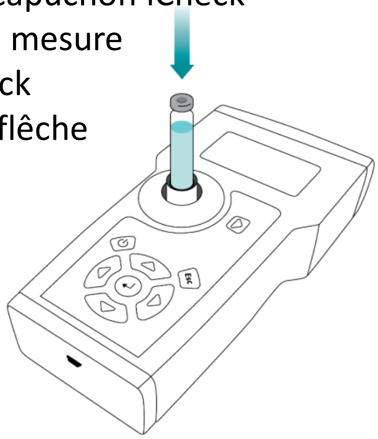

5. Mesurer

- Appuyez encore une fois sur la flêche verte pour commencer l’analyse
- Ré-positionner le flacon 4 fois (« next position »)
- Mettre capuchon, et rappuyer sur flêche verte
- Noter le résultat en mg/L

6. Documenter

- Noter les pesées sur la fiche
- Noter le résultat sur la fiche*
- Vérifiez que toutes les fiches sont au complet!!

* Si vous savez d’avance que la teneur en iode est supérieur à 50 ou 60 ppm, vous diluez les 10 g dans 100 ml d’eau au lieu de 50 ml. Egalement, si le résultat d’un sel inconnu indique une forte teneur en iode, répéter l’analyse avec la dilution 10 g dans 100 ml.

ID-ERTK (Inde)

1. Préparer l'échantillon

- Peser EXACTEMENT 10 g de sel et noter le poids dans votre documentation.
- Ensuite, ajouter EXACTEMENT 50 ml d'eau. Notez le poids exact, et dissolvez le sel.
- En cas d'impurités, filtrer la solution
- S'il s'agit du gros sel, cassez-le avec un mortrier/coupelle

2. Pré-chauffer l'appareil ID-ERTK

- Insérer la cuvette rempli d'eau
- Allumer l'appareil, il marque 'ST'
- Une fois prêt, il marque '00'
- L'appareil a été calibré et est prêt à usage

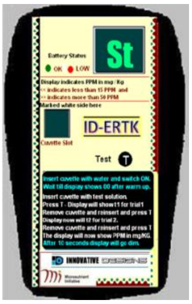

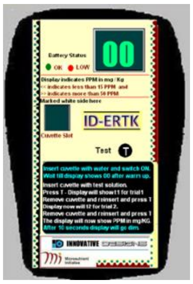

3. Préparer la solution test

- Pipetter1 mL d'échantillon dans la cuvette
- Ajouter 1,5 mL de solution acide (ACIDE)
- Ajouter 0,5 mL de solution KI (Sol KI)
- Ajouter 1,0 mL de solution amidon (AMID)
- Mélanger la solution en ren-versant lentement la cuvette
- Mesurer dans <1minute après mélange!

4. Insérer la cuvette

- Ne pas toucher la partie transparente de la cuvette!
- Insérer la cuvette avec le couvercle
- Vérifier direction: côtés transparents verticalement
- Appuyer sur 'test'
- L'écran montre 't1' pour la 1ère mesure
- Enlever et ré-insérer, appuyer 'test'
- L'écran montre 't2'
- Enlever, ré-insérer, appuyer 'test'
- Après 't3', le résultat s'affiche

5. Lire le résultat

- Si le résultat est entre 15 -50 ppm, la teneur s'affice directement en PPM
- '[[' signifie <15 ppm
- ']]' signifie > 50 ppm
- En cas ou le résultat indique > 50 ppm, répéter l'échantillon en prenant la même quantité de sel, mais double quantité eau → après lecture du résultat, multiplier par 2 afin d'indiquer la bonne concentration

6. Documenter

- Noter les pesées sur la fiche
- Noter le résultat sur la fiche
- Vérifiez que toutes les fiches sont au complet!!

iReader

Peser EXACTEMENT 1 g de sel + 5 mL d'eau

↓ Agiter pour dissoudre le sel

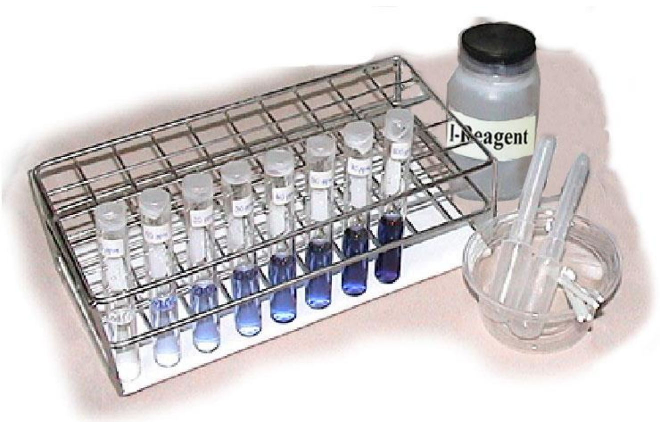

Ajouter 0,55 mL de la solution + 3 mL du I-réactif (I-reagent), soit 2x remplir la pipette dans un tube pyrex

↓ Bien agiter, laisser reposer 5 minutes

En présence d'iode, la solution devient bleue; mesurer la concentration d'iode en PPM de l'ecran de l'appareil

Procédure pour manipuler le I-Reader:

1. Appuyer sur le bouton 'ON/OFF pour allumer l'appareil
2. Insérer un 'blanc' (tube pyrex contenant de l'eau distillée), appuyer '2'
3. Appuyer '1', insérer tube d'échantillon dans la machine, appuyer encore une fois sur '1'
4. Noter la concentration sur l'écran en PPM
5. Répéter les pas 3 et 4 pour d'autres échantillons d'une même série

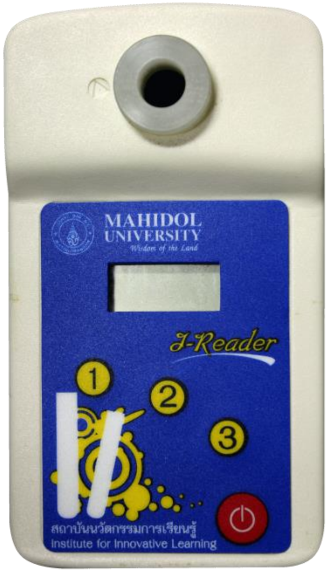

saltPAD

Comment utiliser le saltPAD pour mesurer la teneur en iode dans du sel

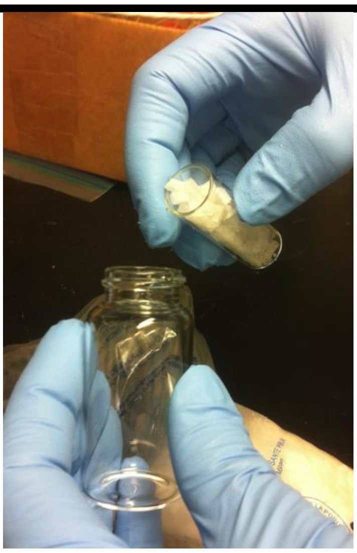

1. Peser 3.25 g sel.*

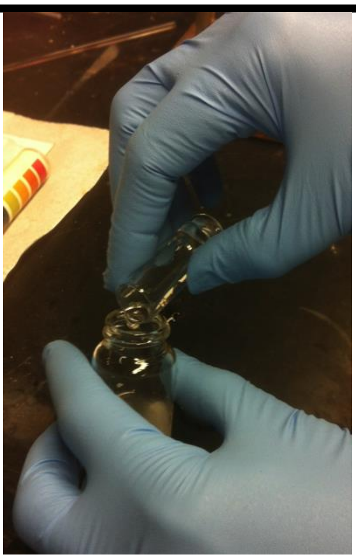

2. Ajouter 15 g eau

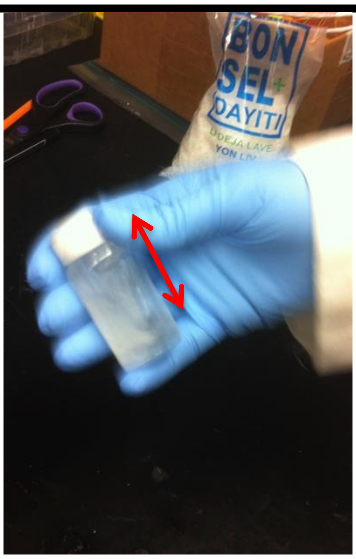

3. Agitez pour dissoudre complètement

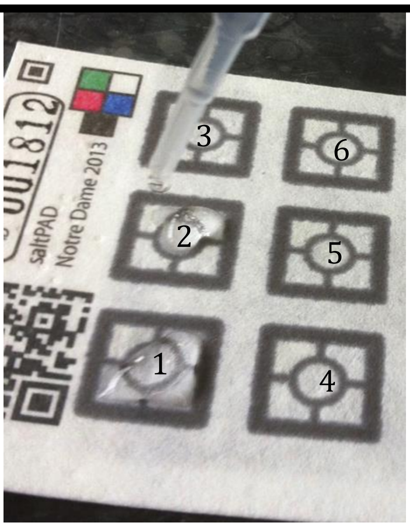

4. Appliquer 3 gouttes (125 μL) de solution à chaque carré dans l'ordre montré. S'assurer que toutes les 5 parties dans chaque carré sont mouillées.

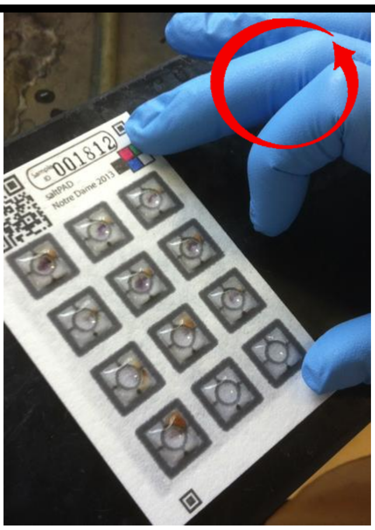

5. Agiter gentiment la carte en cercles pendant 3 minutes.

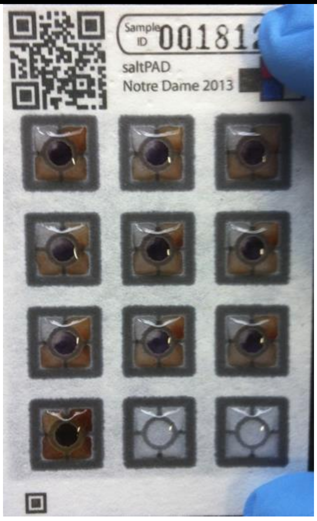

6. Comparer résultats aux images standard, et prendre une photo selon les instructions sur la page suivante.

WYD

1. Matériel supplémentaire requis

- Solution A
- Solution B
- Vérificateur d'iode WYD
- Batteries ou source électrique
- Cuvettes
- Verre de Gray
- Balance de précision (0,01g)
- Tubes de 50ml,
- Chronomètre
- Pipette de 5ml et 10ml

2. Pré-chauffer l'appareil WYD

- Brancher l'appareil ou mettre des batteries
- Appuyer sur ON
- Laisser l'appareil se chauffer pendant 10 minutes
- Si l'appareil est resté longtemps (plus d'une semaine) sans fonctionner, prolonger le temps de chauffage

3. Calibrage du WYD

- Réglage du point Zéro:
- Prendre cuvette et remplir avec eau pure
- Insérer la cuvette dans le bon sens
- Appuyer sur le bouton d'auto calibration (O), la valeur (0,0) apparaît
- Verre de Gray:
- Insérer le verre de Gray
- Ajuster la mesure à 50 avec boutons ^ ou ˅

4. Préparer la solution test

- Peser 1±0,05g d'échantillon bien mélangé
- Transférer cette masse de sel dans un tube de 50 ml
- Ajouter 10ml d'eau distillée dans le tube
- Ajouter 2ml de la solution A
- Ajouter 2ml de la solution B
- Ajouter de l'eau distillée dans le tube jusqu'à 50ml
- Bien secouer la solution

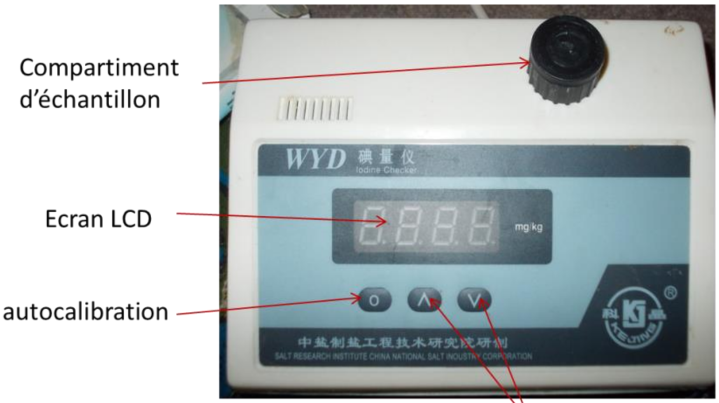

5. Mesure

- Ne pas toucher la partie transparente de la cuvette!
- Insérer la cuvette
- Vérifier direction: côtés transparents horizontalement
- La teneur s'affiche directement en PPM

6. Documenter

- Noter les pesées sur la fiche
- Noter le résultat sur la fiche
- Vérifiez que toutes les fiches sont au complet!!
